# Psychometric Assessment of Shortened Mental Toughness Questionnaires (MTQ): Factor Structure of the MTQ-18 and the MTQ-10

**DOI:** 10.3389/fpsyg.2019.01933

**Published:** 2019-08-21

**Authors:** Neil Dagnall, Andrew Denovan, Kostas A. Papageorgiou, Peter Joseph Clough, Andrew Parker, Kenneth Graham Drinkwater

**Affiliations:** ^1^Department of Psychology, Manchester Metropolitan University, Manchester, United Kingdom; ^2^School of Psychology, Queen’s University Belfast, Belfast, United Kingdom; ^3^Department of Psychology, University of Huddersfield, Huddersfield, United Kingdom

**Keywords:** mental toughness, psychometric validation, MTQ-18, MTQ-10, life satisfaction

## Abstract

The 18-item Mental Toughness Questionnaire (MTQ-18) is a brief, widely used measure of mental toughness. The MTQ-18 derives from the longer MTQ-48, which comprises four independent but correlated factors (challenge, commitment, control, and confidence). Despite sampling items from across MTQ-48 dimensions, the MTQ-18 (as intended) provides a global, unidimensional score. Researchers have recently developed a further abridged version of the MTQ-18, the MTQ-10, which has demonstrated promising psychometric performance. The current paper assessed the factorial structure, reliability, predictive validity and invariance of the MTQ-18 and MTQ-10 in a sample of 944 students from English independent schools (year 11, aged 16 years). Respondents completed the MTQ-18 items online alongside the Satisfaction with Life Scale. Confirmatory factor analysis revealed that the MTQ-10 was a superior general measure, because the MTQ-18 possessed additional variance to that accounted for by an unidimensional solution. Additionally, the MTQ-10 evidenced higher factor loadings and demonstrated better data-model fit. Tests of concurrent validity revealed the MTQ-10 was a stronger predictor of well-being (life satisfaction). Both the MTQ-18 and MTQ-10 demonstrated gender invariance at the configural, metric, and scalar level. Overall, although the MTQ-18 was a psychometrically acceptable measure, the MTQ-10 was a superior unidimensional measure of MT.

## Introduction

Since the 1980s, the concept of mental toughness (MT) has received considerable academic interest (Loehr, 1982; Luszki, 1982). The construct arose from work with elite athletes. Accordingly, Loehr (1982, 1986) defined mental toughness (MT) as stress tolerance and the ability to maximize performance (i.e., the capacity to perform consistently toward the upper range of one’s skills and talents regardless of circumstances) (Loehr, 1994).

Inherent within this conceptualization is ownership of key psychological characteristics. These include the capability to deal with adversity, the ability to thrive under pressure, self-belief, control, resilience, persistence, and superior mental skills (Crust, 2008). Recent reviews by Gucciardi (2017) and Lin et al. (2017) provide an overview of the theoretical development of MT and discuss important methodological issues, which have significantly influenced the construct’s development (see also Gucciardi, 2018). Despite ongoing academic debates about the nature and applicability of MT, it has become a prevailing concept within positive psychology (Rusk and Waters, 2013; Gucciardi, 2017).

In a general context, MT serves as an umbrella term to denote enabling psychological resources across a range of achievement contexts that promote positive mental health (Lin et al., 2017; Drinkwater et al., 2019; Papageorgiou et al., 2019a, b). Such universality can give rise to terminology that lacks a sound empirical basis, is theoretically vague, and contributes to conceptual obfuscation. Noting issues with the definition of MT, Gucciardi (2017) proposed an informed, contemporary delineation. This outlines MT as “a state-like psychological resource that is purposeful, flexible, and efficient in nature for the enactment and maintenance of goal-directed pursuits” (p. 18). Gucciardi’s (2017) characterization recognizes both the traditional roots of MT and its contemporary application to a range of settings. These include sport (Meggs et al., 2018), education (Gerber et al., 2013a; St Clair-Thompson et al., 2015; Haghighi and Gerber, 2018), occupational (Marchant et al., 2009), and health (Coulter et al., 2010; Clough and Strycharczyk, 2012; Brand et al., 2014; Sadeghi Bahmani et al., 2016a; Kruger, 2018).

Concomitant with the absence of a consensually agreed definition, other major concerns have stimulated intense academic debate. Issues center predominately on dimensionality (unidimensional vs. multidimensional), contextual variation (applicability to general vs. context-dependent situations), and dispositional nature (trait vs. state-like). These are important factors to acknowledge because lack of consensus restricts the generalizability of extant findings (Gucciardi et al., 2015) and questions the psychometric integrity of MT measurement instruments. Indeed, unless scales derive from a strong, established research base and demonstrate construct validity there can be no confidence in the legitimacy of reported outcomes (Gucciardi, 2018).

Insofar as researcher preference has informed measurement development, the failure to reach conceptual agreement has undermined the psychometric assessment of MT. The existence of myriad definitions of MT and the advance of various measures evidence this. In this context, scales fall into two broad categories: global (i.e., Mental Toughness Questionnaire, Tiwari and Sharma, 2007) and context specific (i.e., The Sports Mental Toughness Questionnaire, Sheard et al., 2009; Military Training Mental Toughness Inventory, Arthur et al., 2015; Gucciardi, 2018).

For these reasons, it is imperative that researchers establish the psychometric credibility of MT measures before employing them. This is especially true of the Mental Toughness Questionnaire 18-item (MTQ-18 or MT-18) which, despite limited psychometric verification, has featured in a number of peer-reviewed papers (see Table 1 for an indicative list). Explicit concerns are an absence of key details (i.e., rationale for scale, processes involved in item selection, and reporting of measurement properties) (see Clough et al., 2002).

**TABLE 1:** Indicative published studies using shortened mental toughness questionnaire measures (MTQ-18 and MTQ-10).

**Authors**	**Study examined**	**Sample**
Brand et al., 2015a	Whether sleep at kindergarten level predicted sleep and psychological functioning in adolescence.	Adolescents aged 14 years (*SD* = 1.30) (*N* = 37)
Brand et al., 2015b	The relationship between perfectionism and self-reported insomnia severity (controlling for stress and emotion regulation).	Young adult students (*Mage* = 23.87 years, *SD* = 1.93) (*N* = 346).
Brand et al., 2017	Association between vigorous physical activity and restoring sleep, psychological functioning, mental toughness, and male gender.	Early to mid-adolescence (*Mage* = 13.37 years, range = 11–16) (*N* = 1361).
Crust et al., 2010	Mental toughness in an English Premier League academy.	Male football players aged between 12 and 18 years (*N* = 112).
Delaney et al., 2015	Relationships between the MT-18, Big Five personality factors, behavioral inhibition system (BIS), behavioral activation system (BAS), and directed forgetting.	Undergraduates (*N* = 120).
Gerber et al., 2013a	Association between mental toughness and stress resilience.	Vocational school students (*Mage* = 17.86 years) (*N* = 865).
Gerber et al., 2015	Association between burnout and mental health, and tested whether recommended levels of moderate-to-vigorous physical activity attenuated the burnout and mental health relationship.	Vocational school students (*Mage* = 18.10 years, *SD* = 1.20 (*N* = 56).
Gerber et al., 2018	Presence of clinically relevant symptoms of burnout and depression, and possible interaction of perceived stress and mental toughness in the prediction of burnout and depressive symptoms.	Young elite athletes (*Mage* = 16.82 years, *SD* = 1.44) (*N* = 257).
Godlewski and Kline, 2012	Voluntary turnover in Canadian forces recruits.	New military recruits, males (*Mage* = 23.52 years, *SD* = 5.05) (*N* = 459).
Hardy et al., 2014	The incremental predictive validity of trait-based and domain mental toughness scores in the context of learning a complex computer task.	Young-adult males (*Mage* = 19.33, *SD* = 1.77) attending university (*N* = 120)
Kruger, 2018	Mental toughness as a predictor of suicidality in university students.	UK university students (*Mage* = 27.16 years, *SD* = 9.31) (*N* = 113)
Lang et al., 2019	Relationships between self-reported physical activity and personal beliefs about sufficient physical activity are associated with sleep and psychological functioning.	Vocational school students (*Mage* = 17.98 years, *SD* = 1.36 years) (*N* = 864)
Levy et al., 2006	Relationship between mental toughness, sport injury beliefs, pain and adherence to an injury rehabilitation program.	Athletes from private physiotherapy clinics (*Mage* = 32.50 years, *SD* = 10.20) (*N* = 70).
Meggs et al., 2018	Relationships between flow, mental toughness, and subjective performance perception in triathletes.	Triathletes (*Mage* = 28.81 years, *SD* = 3.45) (*N* = 114)
Nicholls et al., 2016	The degree to which coaching behavior in relation to shaping motivational climate influenced the development of mental toughness.	Athletes (*Mage* = 18.60 years, *SD* = 4.60 years) (*N* = 290)
Papageorgiou et al., 2018^∗^	Longitudinal association between MT, narcissism and achievement.	Students ages ranged between 14 and 21 (*N* = 339)
Sabouri et al., 2016	Dark Triad traits in relation to mental toughness and physical activity in young adults.	Adults (*Mage* = 29.0 years, *SD* = 6.58) (*N* = 341)
Sadeghi Bahmani et al., 2016b	Dark Triad traits in relation to mental toughness and physical activity.	Young adults (*Mage* = 29.00 years, *SD* = 6.58) (*N* = 341)

*^∗^Used the 10-item version of the MTQ-18.*

The MTQ-18 is a shortened version of the Mental Toughness Questionnaire 48-item (MTQ-48) (Clough et al., 2002), which is one of the most prevalently used measures of MT. The MTQ-48 assesses total MT and comprises four dimensions: challenge, commitment, confidence (subdivided into two components; interpersonal and own ability) and control (partitioned into two components; emotional and life). Horsburgh et al. (2009) offer support for the MTQ-48’s factorial structure. The MTQ-48 is a widely used measure of mental toughness that possesses established psychometric properties. Specifically, the measure has established internal and test–retest reliability (Levy et al., 2006; Nicholls et al., 2008; Crust, 2009; Gerber et al., 2013a, 2015, 2018). Furthermore, Clough et al. (2002) provide evidence for MTQ-48 construct validity via significant relationships with related measures (i.e., optimism, self-image, satisfaction with life, self-efficacy, and trait anxiety). Gerber et al. (2018) has previously examined the factorial structure of the MTQ-18. Clough et al. (2002) also report criterion validity; participants with self-reported high MT provided lower rating of exertion during a 30 min physically demanding cycling task.

The MTQ-18 uses items drawn directly from the MTQ-48. The justification for the scale is that its brevity makes it highly accessible for end-users. This is advantageous when testing time is restricted, assessment of MT occurs within a large psychological battery of test/measures and fatigue is a potential issue, and/or the participant group is subject to cognitive limitations (i.e., younger participants have shorter attention spans and are more prone to distractions). Accordingly, the MTQ-18 provides a brief, easy to administer/score global measure of MT. Psychometric support for the MTQ-18 frequently references the fact that the scale correlates strongly (*r* = 0.87) with the MTQ-48, which is a well-established measurement instrument (Clough et al., 2002; Nicholls et al., 2016). Beyond this basic analysis, there is little discrete psychometric information regarding the psychometric performance of the MTQ-18. This represents a significant gap in the literature.

One of the earliest studies to provide psychometric details about the MTQ-18, Levy et al. (2006), reported an adequate level of internal reliability (Cronbach’s alpha, α = 0.65) (see Taber, 2018 for a detailed description of alpha values). Crust et al. (2010) (α = 0.69) and Lang et al. (2019) (α = 0.70) observed similar reasonable alpha values. Gerber et al. (2013a, 2015, 2018) in a series of papers (α = 0.70, 0.77, and 0.75 respectively) and Sabouri et al. (2016) (α = 0.80) reported satisfactory internal reliability. Finally, Brand et al. (2015a, b, 2017) (α = 0.94, 0.91, and 0.91 respectively) and Sadeghi Bahmani et al. (2016b) (α = 0.92) observed excellent internal reliabilities within their studies.

Of studies reporting reliability, only the Crust et al. (2010) paper provides details on test–retest reliability. This study used a small, limited sample of 21 academy football players, and found the MTQ-18 was highly stable across a 3-month interval (intraclass correlation > 0.95). Other studies using the MTQ-18, have unfortunately failed to provide comprehensive psychometric information on the measure (i.e., Hardy et al., 2014; Delaney et al., 2015).

Since the MTQ-18 is a truncated version of the MTQ-48 and there is, currently, little literature on the MTQ-18, it is necessary to consider briefly some of the key assumptions underpinning the parent measure. This examination is necessary because debates around the soundness of the MTQ-48 question assumptions underpinning the MTQ-18 and potentially undermine the scale’s presumed psychometric integrity. At a conceptual level, the MTQ measures derive from the delineation of MT as a resistance resource or defense against the effects of stress (Crust and Keegan, 2010), which facilitates coping via production of appropriate attitudes, values, cognitions, and emotions (Nicholls et al., 2011). From this perspective, at a general level, MT moderates the negative effects of stress. Explicitly, it provides individuals with the capacity to deal with pressures and challenges (Clough et al., 2002). Previous work has documented the validity of the stress-moderating function of mental toughness (Gerber et al., 2013a, b, 2018; Haghighi and Gerber, 2018).

A key concern with MTQ measures is dimensionality. The authors of the MTQ-48 contend that the scale comprises four dimensions (Commitment, Challenge, Control, and Confidence) (Clough and Strycharczyk, 2012). Commitment or “stickability” is perseverance and the ability to carry out tasks successfully, despite problems/obstacles. Challenge designates the degree to which individuals see challenges as opportunities for self-development. Control denotes the extent to which the individual believes they have influence over their life (the external environment) and emotions (internal states). Finally, confidence embodies self-belief to complete successfully tasks, particularly confidence in abilities (individual qualities) and interpersonal confidence (being assertive and less likely to be intimidated in social contexts). To date 56 published papers have included the MTQ-48. The majority of these (*n* = 43) have reported the Four C dimensions alongside an overall mental toughness (e.g., Crust and Azadi, 2010). The remaining 13 studies have reported only the global score (e.g., Jackman et al., 2016).

Despite comprising items sampled from each of the MTQ-48 dimensions (Challenge, 3-items; Commitment, 3-items; Control, 5-items; and Confidence, 7-items) the MTQ-18, consistent with the measures design, provides only an overall, unidimensional MT score (Clough et al., 2002). An exception to this was a study by Godlewski and Kline (2012). The authors, for the purposes of structural equation modeling, extracted factors corresponding to the four C dimensions using a principal axis factor analysis using varimax rotation. The factors comprised the highest loading items in each dimension and demonstrated sufficient (Confidence, α = 0.57; Challenge, α = 0.59; and Commitment, α = 0.63) to good internal reliability (Control, α = 0.78) (Taber, 2018).

Within the current literature, there is no explicit justification for why the multidimensional MTQ-48 should give rise to the abridged, unidimensional MTQ-18 (Gucciardi, 2017). This discrepancy thus requires further investigation, especially in light of the fact that other shorter measures often retain dimensionality whilst sampling less construct breadth. For instance, the Big Five Inventory extra-short form (BFI-2-XS) covers all aspects of personality yet contains only 15-items (Soto and John, 2017). Common item variance arising from MTQ-48 dimensionality may produce a less than optimal unidimensional solution. Explicitly, within the MTQ-18 this may represent a general factor indexed by all items that also possesses elements of multidimensionality originating from item parcels that tap similar subject content domains (i.e., MTQ-48 subscale membership) (Reise et al., 2010). Despite issues of dimensionality, global MTQ-48 and MTQ-18 correlate highly.

The lack of information relating to dimensionality within the MTQ-18 resonates with current debates concerning the structure of the MTQ-48. Clough and colleagues produce results that support the factorial validity of the Four C’s Model (Perry et al., 2013, 2015), as do other researchers (e.g., Horsburgh et al., 2009), whereas other researchers question their interpretation (Gucciardi et al., 2012, 2013). Particularly, critics report poor model fit for factors (i.e., several poorly loading items). Indeed, subsequent independent confirmatory factor analysis (CFA) and exploratory structural equation modeling have found that the hypothesized correlated 4-factor model did not produce good data fit in athlete and workplace samples (Gucciardi et al., 2012); misfit was evident at both the global (i.e., model-data congruence) and local (i.e., pattern of factor loadings) levels. Based on these observations and other research, Gucciardi et al. (2015) contend that MT is a unidimensional concept that plays an important role in performance, goal progress, and thriving despite stress.

Noting the MTQ-48 dimensionality issue and the fact that well-regarded studies have employed the MTQ-18, the present paper examined the factorial structure of the MTQ-18 measure. Expanding upon this, analysis also evaluated the psychometric properties of a newly developed 10-item version of the MTQ (MTQ-10) (Papageorgiou et al., 2018). Establishing this measure was necessary in order to provide a succinct, global assessment of Mental Toughness in the context of utilizing an additional large questionnaire battery. Papageorgiou et al. (2018) used this in a recent study examining longitudinal associations between narcissism, mental toughness, and school achievement. The MTQ-10 derived from analysis of the MTQ-48 via selection of the highest line-adding items in each of the four dimensions (i.e., challenge, commitment, control, and confidence). Specifically, this resulted in 12 items (three items relating to each dimension). Initial CFA revealed that two items loaded poorly on a general factor. Subsequent removal of these items resulted in the 10-item, unidimensional scale comprising two items from both challenge and commitment, and three items from both control and confidence. Cross-lagged analyses across two data collection waves by Papageorgiou et al. (2018) demonstrated that the MTQ-10 was stable over time.

The existence of 18 and 10-item versions of the MTQ provides the opportunity to compare the performance of the two brief scales measures, particularly to assess whether they function effectively as unidimensional measures. In this context, the present study evaluated the psychometric performance of the MTQ-18 and MTQ-10. This was an important topic because it further informs debates around MTQ measures. It is important to attempt to resolve measurement issues because persisting conflicting operationalizations thwart conceptual development and undermine MT as a psychological construct. Additionally, this study establishes the psychometric validity of the shortened measures and in doing so demarcates the parameters of use.

A further stage in comparing the performance of the MTQ-18 and MTQ-10 was to assess the predictive capacity of the scales in relation to an established MT criterion, specifically life satisfaction. Research has consistently documented that higher levels of mental toughness (measured with the MTQ-48 and MTQ-18) are associated with greater levels of life satisfaction (Clough et al., 2002; Gerber et al., 2013a). Life satisfaction offers a suitable index of adjustment and adaptive functioning (Gerber et al., 2013a), and is representative of a range of positive psychology measures (e.g., optimism, Nicholls et al., 2008; self-esteem, Earle, 2006) that are typically related to higher levels of MT. To ensure consistency with the focus of previous research utilizing the MTQ-18 and MTQ-10 (see Table 1 for a summary), the current study used a student sample.

## Materials and Methods

### Participants

The sample comprised 944 Year 11 students who were 16 years of age, drawn from several independent schools within England. Consideration of sample composition revealed that 632 (66%) respondents were male, 307 (32.5%) female, and the remaining 14 (1.5%) preferred not to say. Data collection occurred as part of a project investigating the potential impact of sports participation on resilience and psychological well-being. Head teachers from participating institutions invited eligible pupils to participate. Students who responded participated as volunteers.

### Procedure

Head teachers, via email, invited eligible Year 11s to participate. Prior to undertaking the online measures (hosted by Qualtrics) potential respondents received the study brief. This delineated study aims, purpose, content, and ethical procedures. Consenting respondents demonstrated informed consent by selecting a survey option confirming willingness to participate. Following this, respondents advanced to the study materials. Alongside the measures (specifically MTQ-18, MTQ-10, and SWLS), participants completed a brief demographics section which asked for confirmation of age, school, and preferred gender. Next respondents progressed through to the measures. These included a section on sports participation, but this was not analyzed within the present study. Further instructions asked respondents to work through the measures systematically, respond to all items in an open and honest manner and work at their own pace, and reassured respondents that there were no right or wrong answers. On completing the materials, participants were thanked and received a short debrief reaffirming the study’s purpose and their ethical rights.

### Ethics Statement

The research team gained ethical authorization for the project (The Potential Benefits and Costs of Participation in School Sport: A Cross-Sectional and Longitudinal Study). The study investigated the impact of sports participation on resilience and psychological well-being. Following formal submission, the Director of the Research Institute for Health and Social Change and the Manchester Metropolitan University Faculty of Health, Psychology and Social Care Ethics Committee granted ethical approval.

### Measures

#### Mental Toughness Questionnaire 18-Item (MTQ-18) (Clough et al., 2002)

The MTQ-18 uses a selection of items from the MTQ-48 (three Challenge, three Commitment, five Control, and seven Confidence). Items appear as statements (e.g., “I generally feel in control”) and respondents indicate their level of agreement via a five-point Likert-type scale anchored at 1 = strongly disagree and 5 = strongly agree. Summing of individual item responses produces an overall score. Higher scores indicate greater levels of MT. Please see the Introduction for commentary on established psychometric quality. In this study, the internal consistency of the MTQ-18 was satisfactory, α = 0.82.

#### Mental Toughness Questionnaire 10-Item (MTQ-10) (Papageorgiou et al., 2018)

The MTQ-10 is an abridged version of the MTQ-18; it comprises the highest line-adding items in each of the four dimensions (i.e., challenge, commitment, control, and confidence). For example, “I generally cope well with any problems that occur.” The MTQ-10 like other MTQ measures uses a five-point Likert and provides an overall score of mental toughness. Although MTQ-10 has demonstrated promising psychometric properties, further validation is required (Papageorgiou et al., 2018). Specifically, Papageorgiou et al. (2018) reported good model fit via CFA for a unidimensional solution, adequate composite reliability and Cronbach’s alpha across two time points (Time 1 ρ*c* = 0.77 and Time 2 ρ*c* = 0.73; Time 1 α = 0.76 and Time 2 α = 0.75), in addition to test–retest reliability (0.74). The MTQ-10 evidenced satisfactory reliability in the current study, α = 0.77.

#### The Satisfaction With Life Scale (SWLS) (Diener et al., 1985)

The Satisfaction with Life Scale (SWLS) assesses global cognitive judgments of contentment with life (Diener et al., 1985). The measure consists of five statements: (1) In most ways, my life is close to my ideal; (2) The conditions of my life are excellent; (3) I am satisfied with my life; (4) So far, I have achieved the important things I want in life; (5) If I could live my life over, I would change almost nothing. Participants indicate their degree of agreement using a seven-point Likert scale. Possible responses are 1 = strongly disagree, 2 = disagree, 3 = slightly disagree, 4 = neither agree nor disagree, 5 = slightly agree, 6 = agree, and 7 = strongly agree. Summation of items produces an overall total. High scores indicate greater levels of life satisfaction. The SWLS possesses good psychometric properties. These include construct validity, internal consistency, and test–retest reliability (Pavot and Diener, 2008). Internal consistency was satisfactory for the SWLS in this study, α = 0.85.

### Data Analysis

#### Descriptive Analyses

Data screening for normality occurred prior to considering correlations between study variables. For univariate normality, this considered if skewness and kurtosis scores fell within the recommended range of −2 and +2 (Byrne, 2010). Multivariate normality applied Mardia’s coefficient with a critical ratio, which should fall below 5 (Bentler and Wu, 2005). This study used Cohen’s (1988) conventions to interpret the strength of correlations, with 0.1–0.29 representative of a weak relationship; 0.3–0.49 indicative of a moderate relationship; and 0.50 or larger representative of a strong correlation.

#### Confirmatory Factor Analyses

**FIGURE 1:**
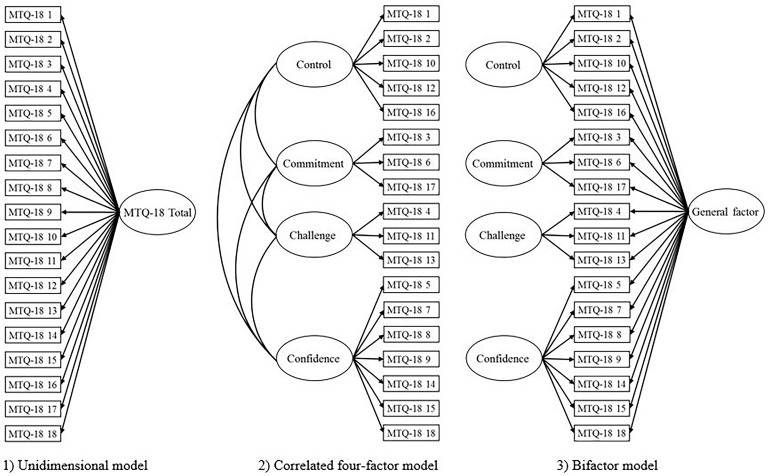
Competing factor models of the MTQ-18.

Subsequent analysis, using Amos 25, examined factor models (1-factor, correlated 4-factor and 4-factor bifactor solutions) for the MTQ-18 and MTQ-10 (Figure 1). The 1-factor model is consistent with previous MTQ-18 literature, and assumes that items load on a single dimension. The 4-factor models assessed the measures in terms of the original MTQ-48 multidimensional approach, which advocates the presence of four latent factors. Particularly, research evidence (e.g., Perry et al., 2013) dictated the allocation of items to subfactors of Commitment, Control, Confidence and Challenge. The 4-factor solution comprised correlated subfactors because this operationalization is consistent with the view of MTQ-48 as a multidimensional measure comprising intercorrelated subfactors (Perry et al., 2013). The bifactor model consisted of the four subfactors in addition to a general Mental Toughness factor. Here, all items loaded onto the general factor as well as the respective subfactors. Bifactor modeling was appropriate given the absence of consensus regarding dimensionality of Mental Toughness and its associated measures (Reise et al., 2010).

The maximum likelihood (ML) method estimated model parameters, and several indices assessed model fit: chi-square, Comparative Fit Index (CFI), Standardized Root-Mean-Square Residual (SRMR) and Root-Mean-Square Error of Approximation (RMSEA). Chi-square in isolation is insufficient to determine the suitability of model fit (Byrne, 2010). Hence, analysis considered also CFI, SRMR, and RMSEA. Consistent with Browne and Cudeck (1993), an acceptable model required SRMR < 0.08, RMSEA < 0.08, and CFI > 0.90. Reporting of RMSEA included the 90% confidence interval (CI). For model comparison, analysis included Akaike’s Information Criterion (AIC), with lower values indicating superior fit.

Consideration of Modification Indices (MI) occurred within each analysis of model fit. Particularly, MI values in excess of 20 were scrutinized (Rossier et al., 2012). Statisticians typically discourage covarying item errors. However, assessment of MI was necessary in this study because some subfactor items were similar in phrasing (Byrne, 2010). As a rule of thumb to assess the significance of factor loadings corresponding to each model, loadings of 0.3 indicate a minimum threshold of acceptance (Hair et al., 2010). This suggests that an item possesses a weak correlation with an extracted factor. Similarly, Hair et al. (2010) assert that the majority of factor loadings should exceed 0.5 to indicate practical significance for a measure. In contrast with 0.3, a loading of 0.5 infers that an item evidences a medium correlation with a factor.

#### Multi-Group Analysis

Multi-group CFA analyzed a progressively restrictive sequence of models to reveal the degree of invariance that existed among the responses of men and women. Specifically, analysis considered invariance of factor structure (configural invariance), factor loadings (metric invariance), and item intercepts (scalar invariance). In addition, using Chen’s (2007) criteria, CFI values should not change by more than 0.01 and RMSEA should not alter by more than 0.015 across the invariance models.

#### Structural Equation Models

The final stage of analysis evaluated the predictive capacity of the MTQ-18 vs. the MTQ-10 by specifying and testing structural equation models. These regressed superior MTQ solutions onto life satisfaction (SWLS), a pertinent and often cited MT outcome (e.g., Crust and Azadi, 2010; Gerber et al., 2013a, 2015).

## Results

### Descriptive Analyses

Kurtosis and skewness fell between −2 and +2 indicating acceptable normal univariate distribution (see Table 2). Mardia’s coefficient was 52.732 (critical ratio = 30.190) for the MTQ-18, and 19.348 (critical ratio = 19.186) for the MTQ-10. These results indicated multivariate non-normality, which can produce standard error biases (Bentler and Wu, 2005). Consequently, CFA analyses utilized ML estimation with bootstrapping (resampled 600 times) to create accurate standard errors alongside bias-corrected *p*-values and confidence intervals (at the 95% confidence level) (Byrne, 2010). Naïve bootstrapping functions effectively when data is non-normal and is a robust alternative to other methods of robust ML estimation (e.g., the Satorra–Bentler chi-square) (Nevitt and Hancock, 2001).

**TABLE 2:** Means, standard deviations, and correlations for all study variables.

**Variable**	***M***	***SD***	**Skewness**	**Kurtosis**	**1**	**2**	**3**	**4**	**5**	**6**	**7**	**8**	**9**	**10**	**11**
(1) MTQ-18 Total	60.176	9.761	–0.271	0.246		0.837^∗∗^	0.646^∗∗^	0.700^∗∗^	0.844^∗∗^	0.932^∗∗^	0.759^∗∗^	0.604^∗∗^	0.649^∗∗^	0.758^∗∗^	0.516^∗∗^
(2) MTQ-18 Control	15.544	3.517	–0.109	–0.184			0.456^∗∗^	0.492^∗∗^	0.561^∗∗^	0.811^∗∗^	0.901^∗∗^	0.391^∗∗^	0.467^∗∗^	0.582^∗∗^	0.403^∗∗^
(3) MTQ-18 Commitment	8.882	2.540	–0.097	–0.422				0.368^∗∗^	0.318^∗∗^	0.630^∗∗^	0.324^∗∗^	0.921^∗∗^	0.333^∗∗^	0.350^∗∗^	0.320^∗∗^
(4) MTQ-18 Challenge	10.785	1.977	–0.241	0.013					0.487^∗∗^	0.660^∗∗^	0.472^∗∗^	0.353^∗∗^	0.834^∗∗^	0.436^∗∗^	0.376^∗∗^
(5) MTQ-18 Confidence	24.965	4.492	–0.536	0.400						0.743^∗∗^	0.551^∗∗^	0.329^∗∗^	0.488^∗∗^	0.800^∗∗^	0.459^∗∗^
(6) MTQ-10 Total	33.835	6.117	–0.235	–0.017							0.814^∗∗^	0.644^∗∗^	0.695^∗∗^	0.817^∗∗^	0.534^∗∗^
(7) MTQ-10 Control	9.585	2.399	–0.125	–0.317								0.304^∗∗^	0.483^∗∗^	0.551^∗∗^	0.377^∗∗^
(8) MTQ-10 Commitment	6.343	1.921	–0.198	–0.593									0.320^∗∗^	0.353^∗∗^	0.336^∗∗^
(9) MTQ-10 Challenge	7.429	1.429	–0.411	0.334										0.444^∗∗^	0.345^∗∗^
(10) MTQ-10 Confidence	10.477	2.364	–0.527	0.092											0.517^∗∗^
(11) Life Satisfaction	19.791	5.500	–0.310	–0.501											

*^∗∗^*p* < 0.001.*

Consideration of zero-order correlations revealed moderate to strong positive relationships among MT totals and subfactors (Table 2). Additionally, moderate to strong positive relationships existed between MT totals and subfactors with life satisfaction.

### Confirmatory Factor Analyses

The 1-factor solution for the MTQ-18 reported unsatisfactory fit across indices: χ^2^ (135, *N* = 944) = 1613.439, *p* < 0.001, CFI = 0.640, SRMR = 0.087, RMSEA = 0.108 (CI of 0.103 to 0.113) (see Table 3). Covarying errors for items 2 and 8, 3 and 6, 7 and 9, 7 and 18, 9 and 18, 12 and 17, and 14 and 15 produced satisfactory fit: χ^2^ (128, *N* = 944) = 542.065, *p* < 0.001, CFI = 0.900, SRMR = 0.055, RMSEA = 0.059 (CI of 0.054 to 0.064). Moreover, this model fitted data significantly better than the unconstrained solution: χ^2^difference (7, *N* = 944) = 1071.374, *p* < 0.001.

**TABLE 3:** Fit indices for alternative measurement models of the MTQ-18 and MTQ-10.

**Model**	**χ^2^**	***df***	**CFI**	**SRMR**	**RMSEA (90% CI)**	**AIC**
**MTQ-18**						
1-factor	1613.439^∗∗^	135	0.640	0.087	0.108 (0.103–0.113)	1721.439
1-factor with correlated errors	542.065^∗∗^	128	0.900	0.055	0.059 (0.054–0.064)	664.065
Correlated 4-factor	1392.327^∗∗^	129	0.692	0.083	0.102 (0.097–0.107)	1512.327
4-factor with correlated errors	805.513^∗∗^	123	0.834	0.063	0.077 (0.072–0.082)	937.513
Bifactor	882.458^∗∗^	117	0.813	0.065	0.083 (0.078–0.088)	1026.458
**MTQ-10**						
1-factor	310.574^∗∗^	35	0.854	0.061	0.091 (0.082–0.101)	350.574
1-factor with correlated errors	128.190^∗∗^	33	0.950	0.037	0.055 (0.045–0.066)	172.190
Correlated 4-factor	191.971^∗∗^	29	0.914	0.048	0.077 (0.067–0.088)	243.971
Bifactor	167.828^∗∗^	29	0.927	0.045	0.071 (0.061–0.082)	239.828

*χ^2^, chi-square goodness-of-fit statistic; *df*, degrees of freedom; CFI, Comparative Fit Index; SRMR, Standardized Root Mean-Square Residual; RMSEA, Root Mean-Square Error of Approximation; AIC, Akaike Information Criterion; ^∗∗^χ^2^ significant at *p* < 0.001.*

The correlated 4-factor model (comprising subfactors of Commitment, Confidence, Control, and Challenge) demonstrated unsatisfactory fit across indices: χ^2^ (129, *N* = 944) = 1392.327, *p* < 0.001, CFI = 0.692, SRMR = 0.083, RMSEA = 0.102 (CI of 0.097 to 0.107). Permitting covariance of errors between items 2 and 16, 7 and 18, 7 and 9, 8 and 9, 9 and 18, and 14 and 15 resulted in better fit on RMSEA and SRMR: χ^2^ (123, *N* = 944) = 805.513, *p* < 0.001, CFI = 0.834, SRMR = 0.063, RMSEA = 0.077 (CI of 0.072 to 0.082). Model fit was significantly better than the unconstrained 4-factor solution: χ^2^difference (6, *N* = 944) = 586.814, *p* < 0.001. However, the model did not reach satisfactory fit across indices.

A 4-factor bifactor model also demonstrated unsatisfactory fit across indices, but SRMR: χ^2^ (117, *N* = 944) = 882.458, *p* < 0.001, CFI = 0.813, SRMR = 0.065, RMSEA = 0.083 (CI of 0.078 to 0.088). Covariance among errors was not permissible for this solution because recommended changes would result in correlating error terms among items belonging to distinct subfactors. A comparison of AIC values suggested that the 1-factor model was the superior solution. This had a lower AIC (664.065) compared with the correlated 4-factor (937.513) and 4-factor bifactor (1026.458) solutions.

An assessment of factor loadings for the MTQ-18 1-factor solution revealed that all items apart from 18 and 7 loaded greater than the minimum threshold of 0.3. Similarly, 33.33% of items loaded above 0.5 (a cut-off to indicate practical significance) (Hair et al., 2010). This suggests that the scale comprises a majority of items that do not evidence practical significance, with two items failing to meet the minimum recommended threshold.

Similarly to the MTQ-18 results, the MTQ-10 1-factor model demonstrated unsatisfactory fit across all indices, but SRMR: χ^2^ (35, *N* = 944) = 310.574, *p* < 0.001, CFI = 0.854, SRMR = 0.061, RMSEA = 0.091 (CI of 0.082 to 0.101). Permitting error covariance between items 2 and 8, and 3 and 6 significantly improved model fit: χ^2^difference (2, *N* = 944) = 182.384, *p* < 0.001. This solution also possessed good fit across indices: χ^2^ (33, *N* = 944) = 128.190, *p* < 0.001, CFI = 0.950, SRMR = 0.037, RMSEA = 0.055 (CI of 0.045 to 0.066).

The correlated 4-factor solution reported satisfactory fit: χ^2^ (29, *N* = 944) = 191.971, *p* < 0.001, CFI = 0.914, SRMR = 0.048, RMSEA = 0.077 (CI of 0.067 to 0.088). In addition, the 4-factor bifactor model indicated suitable overall fit: χ^2^ (29, *N* = 944) = 167.828, *p* < 0.001, CFI = 0.927, SRMR = 0.045, RMSEA = 0.071 (CI of 0.061 to 0.082). Similarly to the MTQ-18 results, error covariance was not permissible for these solutions because recommended changes necessitated correlating error terms among items belonging to discrete subfactors. Assessment of AIC results revealed that the 1-factor solution (AIC of 172.190) demonstrated better data fit than the correlated 4-factor (AIC of 243.971) and 4-factor bifactor (AIC of 239.828) models.

An inspection of factor loadings revealed that all items loaded above the minimum threshold of 0.3, and 50% of items loaded greater than 0.5. These results infer that the scale satisfies the minimum requirements of Hair et al. (2010) overall.

### Multi-Group Analysis

An assessment of gender invariance occurred for MTQ-18 and MTQ-10 superior factor solutions (i.e., the 1-factor models). For the MTQ-18, a test of configural invariance revealed satisfactory fit across RMSEA and SRMR, but not CFI: χ^2^ (256, *N* = 944) = 710.224, *p* < 0.001, CFI = 0.885, SRMR = 0.066, RMSEA = 0.044 (CI of 0.040 to 0.048). The metric invariance test demonstrated similar results. However, the CFI difference (0.001) and RMSEA difference (0.002) were minimal, supporting invariance among the factor loadings. In addition, the scalar invariance test demonstrated an acceptable change in CFI (0.028) and RMSEA (0.004). This indicated invariance at the intercept level.

For the MTQ-10, configural invariance analysis revealed good fit across indices: χ^2^ (66, *N* = 944) = 148.374, *p* < 0.001, CFI = 0.954, SRMR = 0.042, RMSEA = 0.037 (CI of 0.029 to 0.045). In relation to the metric model, the difference in CFI (0.001) and RMSEA (0.002) was minimal, suggesting invariance for the factor loadings. The scalar model also demonstrated minimal change in CFI (0.001) and RMSEA (0.001), indicating invariance among the item intercepts.

### Structural Equation Models

**FIGURE 2:**
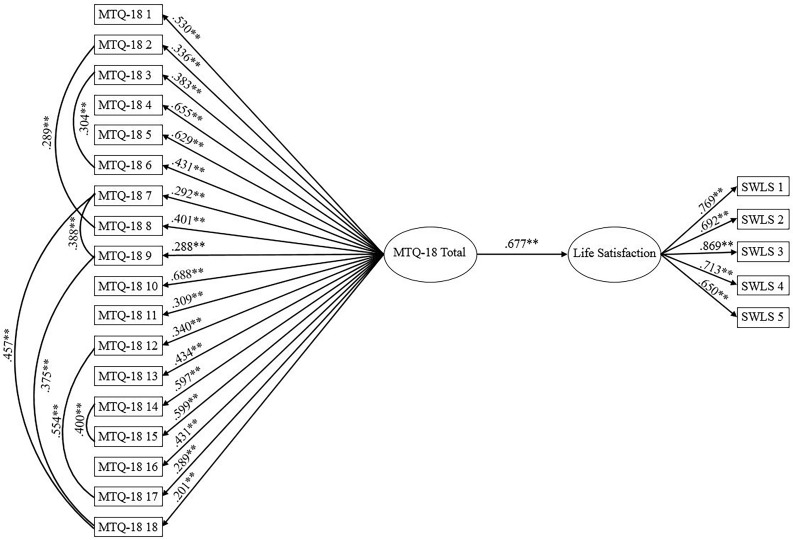
MTQ-18 as a predictor of life satisfaction. Ellipses represent latent variables; measured variables are represented by rectangles; error is not shown but was specified for all variables. ^∗∗^*p* < 0.001 (using bootstrap significance estimates).

A structural equation model using the 1-factor mental toughness solution as a predictor of life satisfaction for the MTQ-18 (Figure 2) indicated satisfactory model fit: χ^2^ (222, *N* = 944) = 863.854, *p* < 0.001, CFI = 0.904, SRMR = 0.053, RMSEA = 0.055 (CI of 0.052 to 0.059).

**FIGURE 3:**
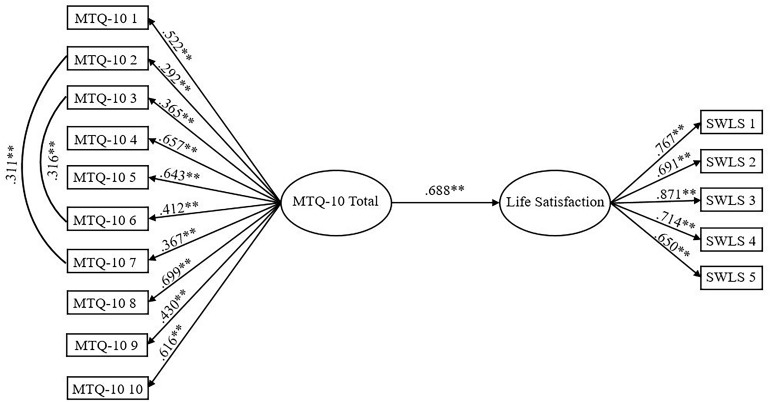
MTQ-10 as a predictor of life satisfaction. Ellipses represent latent variables; measured variables are represented by rectangles; error is not shown but was specified for all variables. ^∗∗^*p* < 0.001 (using bootstrap significance estimates).

Inspection of structural paths revealed that mental toughness significantly predicted life satisfaction, β = 0.677, *p* < 0.001. Replicating the structural equation model for the superior MTQ-10 solution (the 1-factor model; Figure 3) demonstrated good data-model fit: χ^2^ (87, *N* = 944) = 358.296, *p* < 0.001, CFI = 0.939, SRMR = 0.044, RMSEA = 0.058 (CI of 0.051 to 0.064). The structural paths revealed that mental toughness was a significant predictor of life satisfaction, β = 0.688, *p* < 0.001.

## Discussion

Comparison of unidimensional (1-factor) and factorial models (correlated 4-factor and 4-factor bifactor) revealed that single factor models were superior to multidimensional alternatives for both the MTQ-18 and MTQ-10. Of the models tested, the MTQ-10 1-factor model demonstrated best fit. In comparison, the MTQ-18 possessed additional variance to that accounted for by a unidimensional solution. This resulted in the need to correlate multiple item error terms, equaling more than 50% of the scale items.

The additional variance was attributable to the methodological approach used to create the MTQ-18 (Clough et al., 2002). In an attempt to sample construct breadth, the MTQ-18 authors selected high loading items from each of the MTQ-48 subscales (challenge, commitment, control, and confidence). It appears that this approach inadvertently introduced structural contamination arising from 4Cs dimensional resonance; latent item associations weakened the intended unidimensional structure. This results in an adequate global measure that derives from a less than optimal factorial solution. Contrastingly, because the MTQ-10 stems from the highest loading scale items (regardless of factor origin) it remains largely untainted by the underlying MTQ-48 structure.

Overall, analysis indicated that the MTQ-10 was a psychometrically superior global measure to the MTQ-18. Particularly, the MTQ-10 had higher factor loadings and demonstrated better data-model fit. Additionally, the MTQ-10 regression path with the established mental toughness criterion, life satisfaction, was stronger.

The conceptual issue of why the abridged MT measures are unidimensional, whereas the parent MTQ-48 scale is multidimensional, is beyond the remit of this paper. However, it is important to note that despite sampling MTQ-48 subscales, the MTQ-18 best fitted a unidimensional model (Gucciardi et al., 2015; Birch et al., 2017; Vaughan et al., 2018) confirming the author’s assertion that the scale provides a global measure of MT.

Although the MTQ-18 performs less well psychometrically than the MTQ-10, this study indicates that the scale is an acceptable, but less parsimonious, measure of global mental toughness. This outcome is reassuring for studies that have used the MTQ-18 to measure global levels of mental toughness (see Table 1). The MTQ-18 and MTQ-10 were highly correlated with each other. In this context, previous work on the MTQ-48 provides a wealth of background evidence that supports the assertion that the MTQ-18 is valid to the extent that it adequately indexes mental toughness as defined by Clough and colleagues. In summary, conceptual disagreements concerning the precise nature of mental toughness are beyond the scope of the present paper, but theoretically important to note (see recent reviews by Gucciardi, 2017 and Lin et al., 2017).

In terms of performance with an established mental toughness criterion, life satisfaction, both the MTQ-18 and MTQ-10 performed similarly to the MTQ-48. The present study observed correlations between both brief measures and Life Satisfaction in the large range (MTQ-18, *r* = 0.52; MTQ-10, *r* = 0.53). These relationships were comparable to those reported by Crust and Clough (2005) (*r* = 0.56) and Marchant et al. (2009) (*r* = 0.56). Correlations generally further supported the well-established finding that higher levels of MT are associated with life satisfaction (Gerber et al., 2013a).

The present study used a sample of Year 11 (16 years old) students to facilitate direct comparisons with related studies, who have generally used commensurate participant groups (i.e., older school students, undergraduates, and young adults). This paper found that the short MTQ scales were appropriate measures of global MT within young people (16 year olds). Additionally, the MTQ-18 and MTQ-10 demonstrated gender invariance indicating that there was no difference between males and females. Furthermore, Gerber et al. (2018) found that testing the factor structure of the MTQ-18 resulted in acceptable model fit in young elite athletes (Gerber et al., 2018). Whilst these results were encouraging, further work is required to establish whether this is also true of other populations. This will inform key conceptual concerns, which have hindered the development of MT (i.e., contextual variations and temporal stability).

Drawing on the MTQ-48 literature, there is evidence that scale structure varies as a function of contextual variations. Particularly, that the appropriateness of the 4C structure varies as a function of sample. For instance, Birch et al. (2017) observed that the 4Cs model did not apply to student athletes. Similarly, Vaughan et al. (2018) found that the 4-factor model produced poor data fit when applied to elite athletes. Noting these factorial discrepancies, consistent with the concerns of Gucciardi (2018), the authors advocate caution when extrapolating the psychometric properties of the MTQ-18 and MTQ-10. Currently, conclusions should remain within the perimeters of young adult and undergraduate students.

Although the present paper indicates that the two concise measures of MT possess sound psychometric properties, there are important unresolved issues that require further evaluation. One particular concern is temporal stability. Currently, there is only limited evidence to indicate that the MTQ-18 (Crust, 2009) and MTQ-10 (Papageorgiou et al., 2018) possess satisfactory test–retest reliability. It is essential to establish test–rest reliability because this supports the internal validity by demonstrating that measurements obtained by a scale are representative and stable over time. Specifically, the ability to provide consistent scores over time in a stable population (Aaronson et al., 2002).

Establishing that scales possess enduring properties is essential at both measurement and theoretical levels. Knowing the limitations of psychometric tools is vital to appropriate score interpretation. In the case of contextual variations, it is necessary to identify group differences in order to generate appropriate norm groups. Conceptually, examining contextual variations and temporal stability informs the development of MT by offering insights into key theoretical questions. Accordingly, the development of the MTQ-10 will provide valuable insights.

Clough and colleagues (e.g., Crust and Clough, 2005) support the notion that MT is a trait-like dimension (Clough et al., 2012), whereas critics contend that MT lacks stability. Acknowledging this, several MTQ-48-related articles refer to the importance of the role of experiential factors. Principally, the notion that exposure to challenging situations facilitates the development of resources through problem solving (Crust and Clough, 2011; Clough et al., 2016). The MTQ-10 provides a brief, easy to administer measure that lends itself to regular completion. Hence, the MTQ-10 will enable researchers to readily assess temporal stability, investigate the effect of intervening factors (i.e., training), and test MT levels across multiple time points and settings.

Recently, Strycharczyk and Clough (2014) postulated that MT as measured by the MTQ-48 is a ‘plastic’ personality trait (Strycharczyk and Clough, 2014). Plastic in this context signifies that level of mental toughness is malleable. It derives from the observation that MT is ‘trainable’ to the extent that people can learn to adopt non-preferential behaviors. In this context, the short MT measures provide expedient, accessible, and easy to interpret indexes for assessing levels of MT in everyday situations (i.e., sport, educational, and occupational).

## Data Availability

The datasets generated for this study are available on request to the corresponding author.

## Ethics Statement

The research team gained ethical authorization for the project (The Potential Benefits and Costs of Participation in School Sport: A Cross-Sectional and Longitudinal Study). This study investigated the impact of sports participation on resilience and psychological well-being. Following formal submission, the Director of the Research Institute for Health and Social Change and the Manchester Metropolitan University Faculty of Health, Psychology and Social Care Ethics Committee granted ethical approval.

## Author Contributions

ND and AD designed the study. ND was the main author. AD conducted the main analysis and wrote the manuscript. PC and KD organized the data collection and commented on the manuscript drafts. KP provided the specialist advice and performed the editing. AP and KD advised on the manuscript contents.

## Conflict of Interest Statement

The authors declare that the research was conducted in the absence of any commercial or financial relationships that could be construed as a potential conflict of interest.
